# Effect of H_2_O Activity on Zeolite Formation

**DOI:** 10.3390/ma13214780

**Published:** 2020-10-26

**Authors:** Claudia Belviso, Francesco Cavalcante

**Affiliations:** Istituto di Metodologie per l’Analisi Ambientale–CNR, 85050 Tito Scalo, Italy; francesco.cavalcante@imaa.cnr.it

**Keywords:** LTA, liquid nitrogen freezing, conventional freezer process, drying temperature, amorphous ice, crystalline ice

## Abstract

In an effort to understand the effects of H_2_O activity on zeolite formation, we have synthesized LTA zeolite using a combination of freezing processes and varying drying temperatures. Sodium aluminate and sodium silicate were used to form LTA zeolite, according to the IZA (International Zeolite Association) protocol. The synthesis steps were modified by adding the precursor frozen process by a rapid liquid nitrogen (−196 °C) treatment or slow conventional freezer treatment (−20 °C). The samples were subsequently sonicated and then dried at 80 °C or 40 °C. X-ray diffraction (XRD) and scanning electron microscopy (SEM) were performed on the samples immediately after the drying process as well as after 2 weeks and 1 month of aging the solid products. The results indicated that LTA zeolite does not form. The silica-alumina precursor after both freezing processes and after being dried at 80 °C showed the presence of sodalite displaying stable behavior over time. Both sets of samples dried at 40 °C and did not show the presence of zeolite immediately after the drying process. However, after 2 weeks, the liquid nitrogen–frozen precursor was characterized by the presence of EMT whereas zeolites never formed in the −20 °C samples. These results suggest that freezing processes differently control the H_2_O activity during the drying and aging processes in the solid state. Thus, although the precursor chemical composition is the same, the type of zeolite formed is different.

## 1. Introduction

Zeolite Linde Type A (Zeolite LTA or Zeolite A) [Na_12_[(AlO_2_)_12_(SiO_2_)_12_] · 27 H_2_O [1] can be synthesised using a variety of sources including different types of alumina and silica materials [2,3,4,5,6,7,8,9,10,11,12,13]. Many nucleation and crystal-growth mechanisms have been proposed for this type of zeolite [14,15,16,17,18], and many literature data have documented microcrystals zeolite A growth on various substrates to form highly oriented monolayers or multilayers composed of micro crystalline building blocks [19,20,21].

Numerous studies have also been performed to investigate the evolution of this type of zeolite into more thermodynamically stable hydroxisodalite [22,23,24]. In our previous papers, two processes for LTA synthesis were analysed concluding that rapid zeolite crystallization by sonication treatment ensures rapid transformation into more stable sodalite, whereas the slower double-step mechanism of geopolymer transformation into the crystalline phase by a conventional hydrothermal process is responsible for a very slow transformation of LTA into sodalite [25,26].

Zeolites are typically synthesized by a hydrothermal process [27] and, indisputably, the water plays a key role in this process. Water molecules have, in fact, an important structural directing action in zeolite formation [28]. The action of H_2_O molecules in precursor materials and metastable products can also be influenced by their position in restricted geometries (nanopores). Literature data have documented that H_2_O confined in mesopores and nanopores shows properties different from those of common bulk water [29,30,31].

Generally, it is assumed that, under ambient conditions, ice occurs in the stable hexagonal form (ice I_h_) or in the metastable cubic form (ice I_c_). However, in the last few years, ice crystallization in nanopores in the form of intercalated cubic and hexagonal layers has been documented [32,33,34]. Jażdżewska and co-workers [35] demonstrated that ice confined in nanopores having pore diameters of 5.9 nm has cubic sequences interlaced with hexagonal sequences, producing a stacking-disordered ice (ice I_sd_). According to the authors [35], the unique behavior in the nanoscale environment is due to a competition between the H_2_O–wall and H_2_O–H_2_O intermolecular interactions as well as an unspecified size effect.

Many other research data indicate that the properties of H_2_O in pores <2 nm are different from the properties of H_2_O in pores with a larger size, and it is common to consider that the freezing temperature of H_2_O in pores decreases with a decline in pore size [36]. For nanopores with a pore size of 2 nm, water can be supercooled to −90 °C [37,38], and Jannes et al. [39] showed that homogeneous nucleation of structural H_2_O molecules takes places at ~−40 °C inside the microporous structure of zeolites. Bordoninskii and Olov [36] described a multiphase transition of ice structures in natural zeolite (natural powder zeolite composed of 90% clinoptilolite) with pore sizes from 0.2 to 2 nm over a range of temperature from −150 to −100 °C. Some simulation studies carried out using zeolite A indicated that the H_2_O nanoclusters in this type of zeolite are too small to crystallize, thus displaying amorphous ice behavior [29]. The formation of low-density amorphous ice in nano-structural zeolite-template carbon was also documented by Kyakuno et al. [40]. Water (liquid water) “freezing” in zeolite (powder zeolite) is influenced by the type of freezing process used. The vitrification determined by liquid nitrogen is based on a rapid temperature lowering process that does not favor the nucleation of crystal water. Due to this, the technique is largely used to preserve biomaterials without ice or with a very limited amount of ice formation [41,42,43]. The slow cooling due to conventional freezer treatments, instead, determines the crystallization of larger water clusters [44]. In the last few years, the application of ultrasound to liquid freezing has also attracted great interest, and freezing by sonication has been used in many applications such as medical science or food engineering [45,46,47,48]. Literature data have shown that an ultrasound can accelerate mass transfer determining rapid droplet cooling [49]. Sonication causes cavitation with formation of bubbles inside the liquid. These bubbles can affect mass transfer and heat transfer mainly at the liquid surface, thereby facilitating liquid cooling and freezing. Some studies have analysed the variation of ultrasound propagation speed based on the physical properties of a medium that they go through (e.g., ice) [50,51]. This mechanism has also been investigated for crystal ice nucleation in water [52,53,54]. However, according to our knowledge, a study on the effects of an ultrasound on frozen hydrogel-colloidal precursors of zeolite has not been performed.

We performed a preliminary study on the effects of a rapid and a slow freezing treatment (by liquid-nitrogen and conventional freezer, respectively) of zeolite precursor combined with subsequent low (−40 °C) or high temperature (80 °C) drying. The potential effect of brief sonication on frozen samples before the drying processes was also investigated. The study aims to provide insights into the fundamental role of water and its form during zeolite crystallization with the hope of providing new insights into understanding formation mechanisms.

## 2. Experimental Section

### 2.1. Samples Preparation

**Figure 1:**
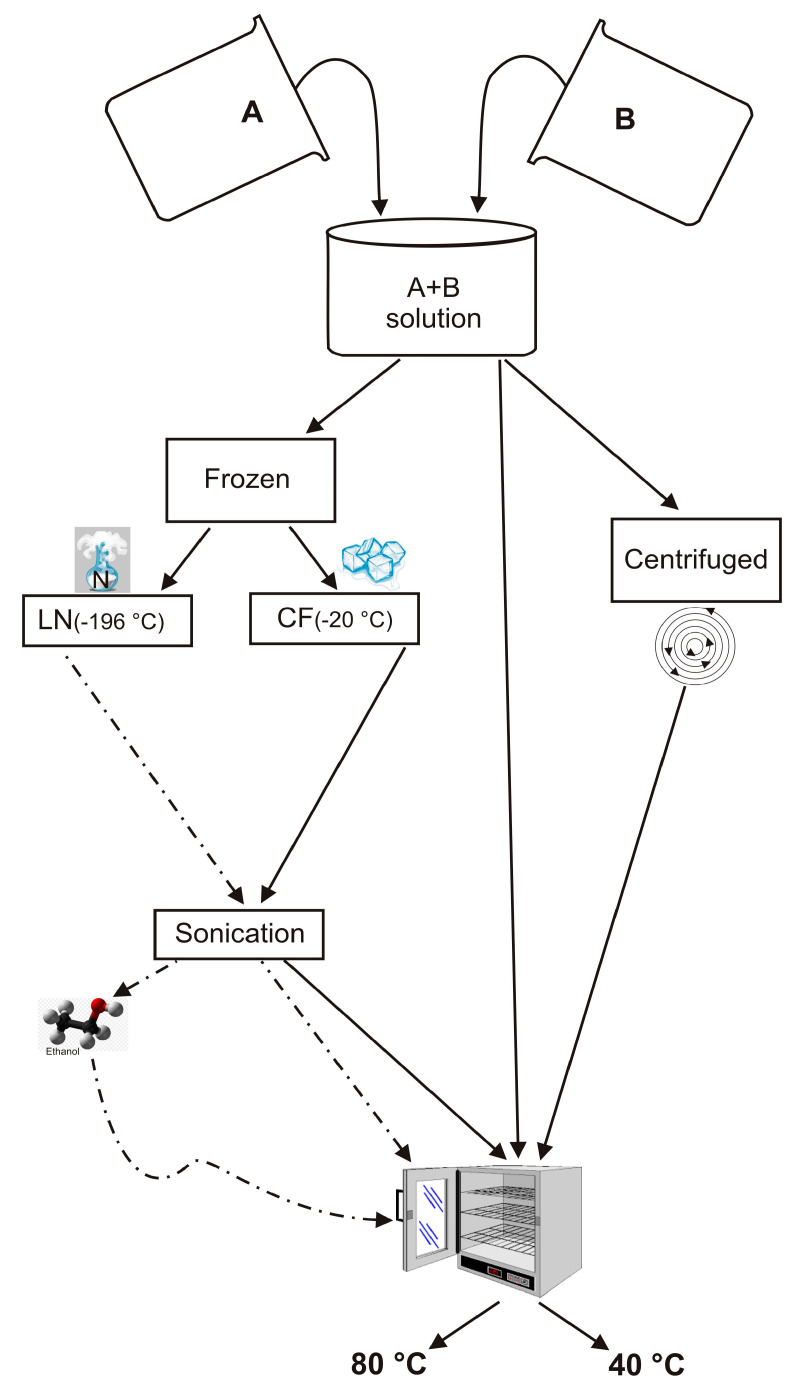
Schematic presentation of the experiments performed.

An NaOH (Sigma Aldrich-Europe, Darmstadt, Germany, 98+% NaOH) solution was prepared by gently mixing 0.723 g of NaOH in 80 mL of distilled water until it is dissolved. Half of this alkaline solution was then used to dissolve 8.3 g of sodium aluminate (Sigma Aldrich-Europe, Darmstadt, Germany, Al (Al_2_O_3_):50–56% Na (Na_2_O):40–45%) (Solution A). In addition, 15.48 g of sodium silicate solution (Sigma Aldrich) were dissolved in the second half (Solution B). Finally, solution A was quickly added to solution B under vigorous stirring. Solutions A and B were prepared according to the IZA protocol and the method proposed by Thompson and Huber [1,55]. However, in this study, the next steps to form LTA zeolite were modified as follows: (i) the resulting suspension (combination of Solution A and B) was divided in two parts, (ii) one part was frozen by liquid nitrogen (~ −196 °C) (LN) for five minutes (rapid freezing treatment) and the other by conventional freezer treatment (~ −20 °C) (CF) for 2 h (slow freezing treatment), (iii) both frozen samples were sonicated using an ultrasonic water bath (240 W, 35 kHz) for 5 min. US temperature was 10 °C, thus melting the samples, iv) both sonicated LN and CF samples were split into two parts and then dried in open vessels at 40 °C and 80 °C for 24 h, respectively. The experiments were also performed by washing LN with ethanol before drying at 40 °C and 80 °C for 24 h (LNE). Finally, additional tests were carried out without any freezing treatment. In detail, the suspension resulting from mixing of Solution A and Solution B was divided into two portions. One was centrifuged and dried at 40 °C and 80 °C for 24 h (ZC), separately. The other portion was directly dried at 40 °C or 80 °C without being centrifuged (Z). The scheme of the experiments is illustrated in Figure 1.

### 2.2. Sample Characterization

The mineralogical composition of all the synthetic products was determined by X-ray powder diffraction (XRD) using a Rigaku Rint 2200 powder diffractometer (Tokyo, Japan) with Cu Kα graphite monochromatized radiation (30 kV–40 mA). XRD profiles were collected in θ–θ geometry over the angular range 3°–70° 2θ, step size 0.02°, and scan-step time of 3 s. The synthetic products were analysed immediately after the drying process (1 day) as well as 2 weeks and 1 month after. The samples were stored at room temperature (about 20 °C). Table 1 summarizes the codes for different samples, according to the processes used for their synthesis. Morphological observation of products was performed by scanning electron microscopy (SEM) using a Zeiss Supra 40 microscope (Oberkochen, Germany) equipped with energy dispersive X-ray analysis (EDX) 

## 3. Results

**Figure 2:**
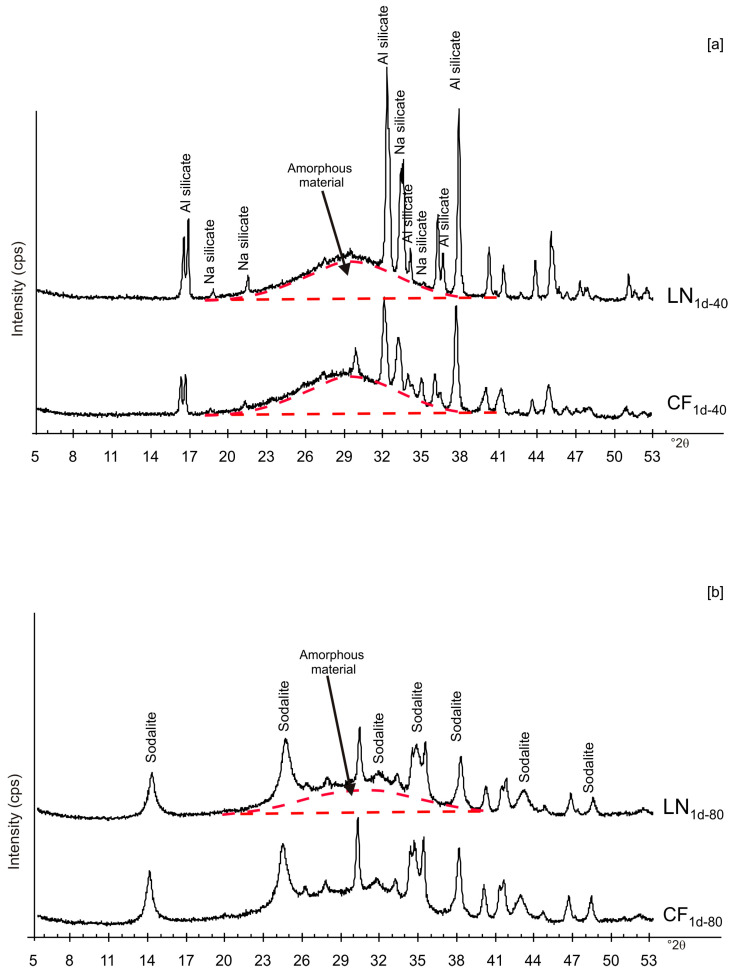
X-ray patterns of CF and LN samples immediately after drying at 40 (**a**) and 80 °C (**b**).

Figure 2 shows X-ray diffraction patterns of the LN_1d_ and CF_1d_ samples immediately after drying at 40 and 80 °C. The samples dried at 40 °C (CF_1d-40_ and LN_1d-40_) are characterized by the presence of aluminium silicate and sodium silicate in addition to amorphous material (Figure 2a). The XRD peaks for the samples dried at higher temperatures (CF_1d-80_ and LN_1d-80_) were mainly indexed to sodalite with minor aluminium silicate and sodium silicate as well as amorphous material (Figure 2b).

**Figure 3:**
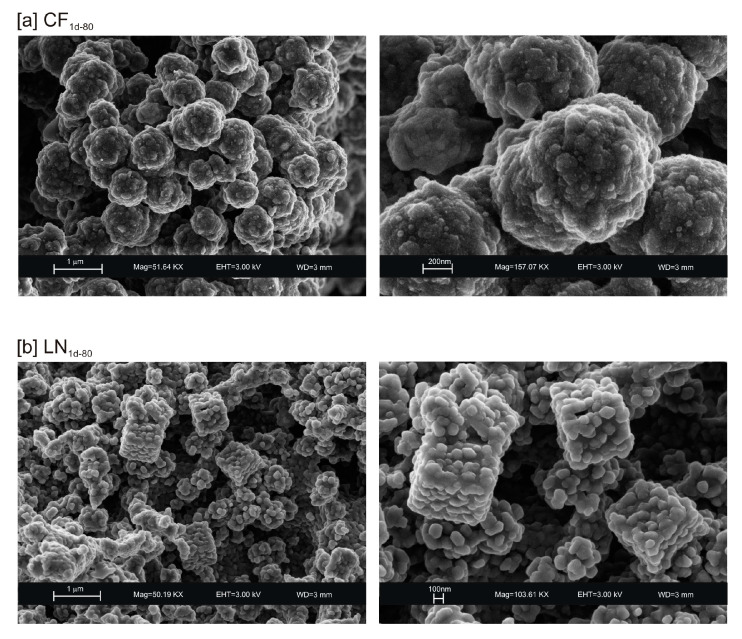
SEM images of: (**a**) CF and (**b**) LN samples after drying 80 °C.

**Figure 4:**
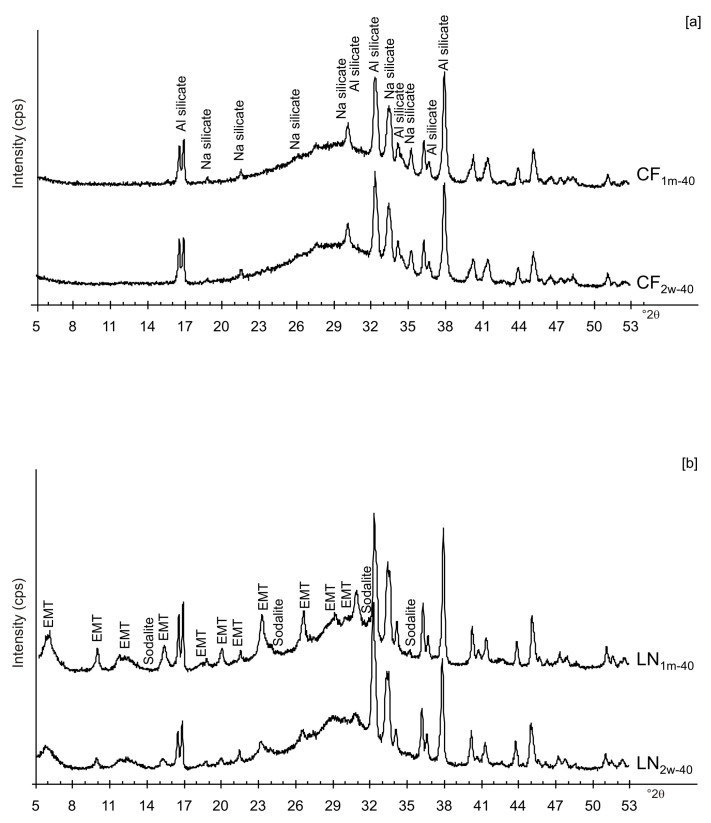
XRPD profiles of: (**a**) CF and (**b**) LN samples dried at 40 °C and analysed after 2 weeks and 1 month.

The presence of spherical nanocrystal aggregates characterizes SEM images of CF_1d-80_ (Figure 3a) whereas the nanocrystals organized to form cubic forms in the LN1d-80 sample (Figure 3b). CF samples dried at 40 °C and analysed after 2 weeks (CF_2w-40_) and 1 month (CF_1m-40_) do not show the presence of any newly formed zeolite (Figure 4a). The peaks are indexed to aluminium silicate and sodium silicate. The XRD pattern of LN dried at 40 °C and analysed after 2 weeks of aging (LN_2w-40_) is, instead, characterized by the presence of EMT zeolite, which increased after 1 month (LN_1m-40_) (Figure 4b and Figure S1).

**Figure 5:**
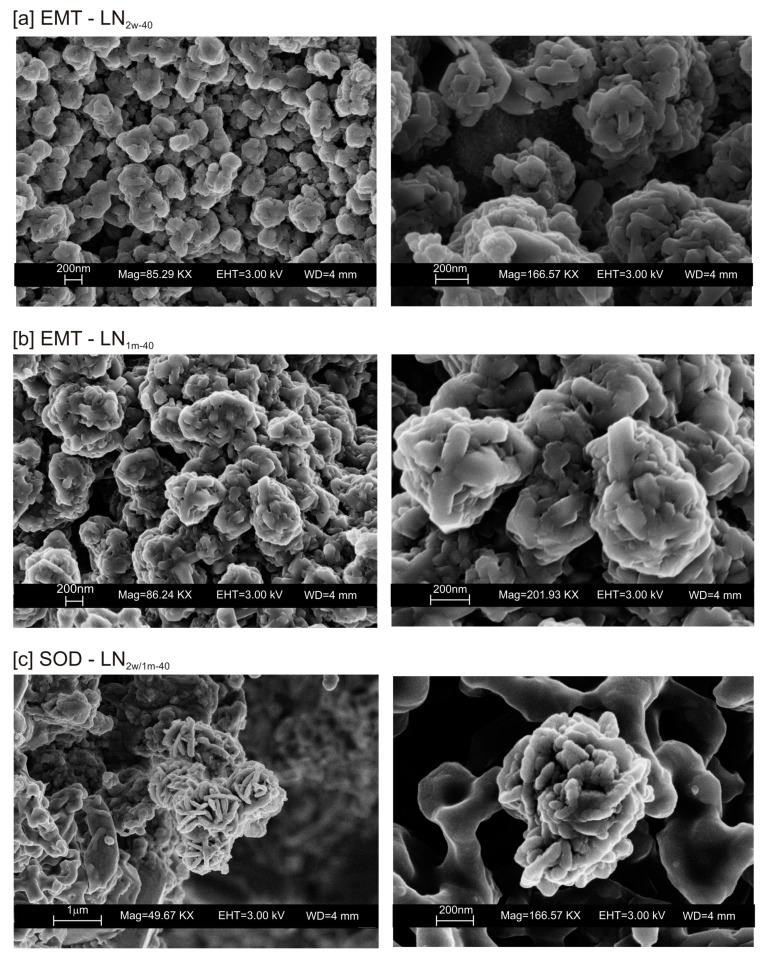
SEM images of EMT zeolite in (**a**) LN_2w-40_ and (**b**) LN_1m-40_. (**c**) Sodalite formed in both LN_2w-40_ and LN_1m-40_.

The typical morphology of this newly formed zeolite is displayed in Figure 5a,b. The presence of other zeolites (e.g., sodalite) was confirmed by SEM (Figure 5c).

**Figure 6:**
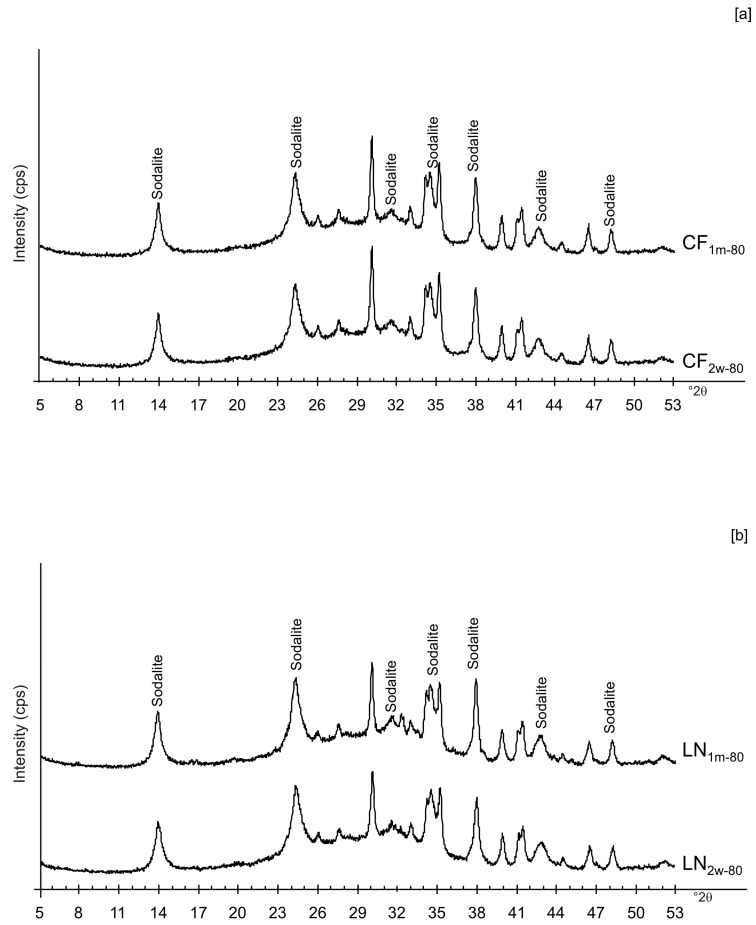
XRPD profiles of: (**a**) CF and (**b**) LN samples dried at 80 °C and analysed after 2 weeks and 1 month.

**Figure 7:**
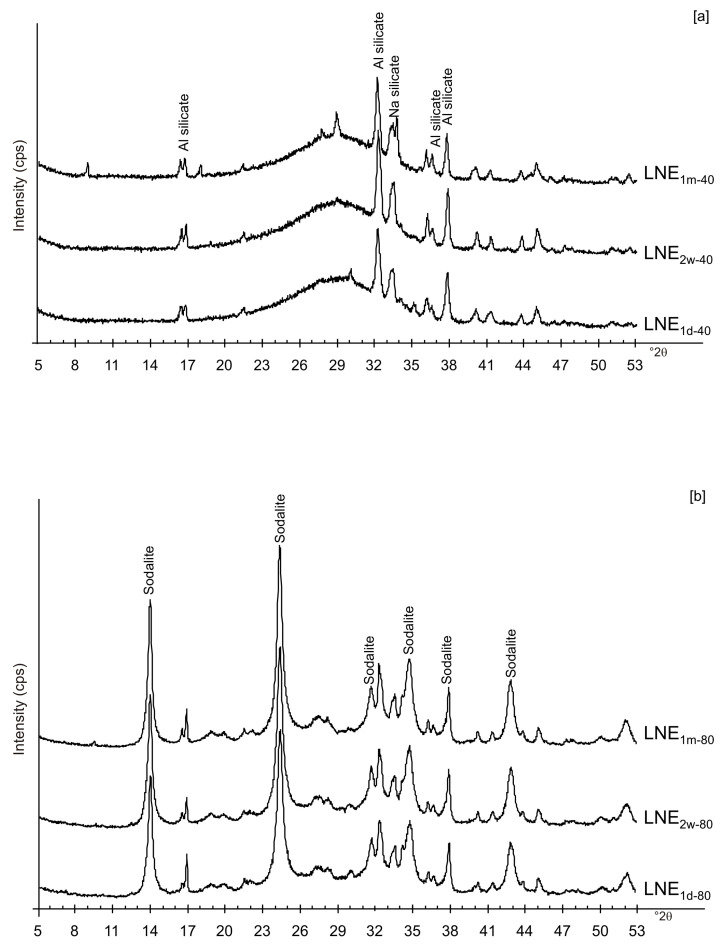
XRD pattern of LN samples after ethanol washing and drying at (**a**) 40 °C and (**b**) 80 °C.

**Figure 8:**
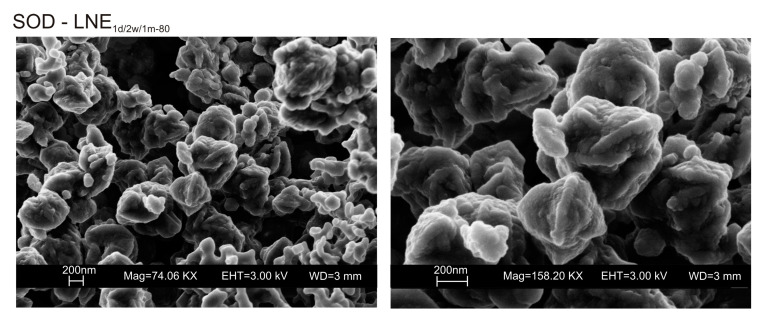
SEM images of sodalite formed in LNE samples.

**Figure 9:**
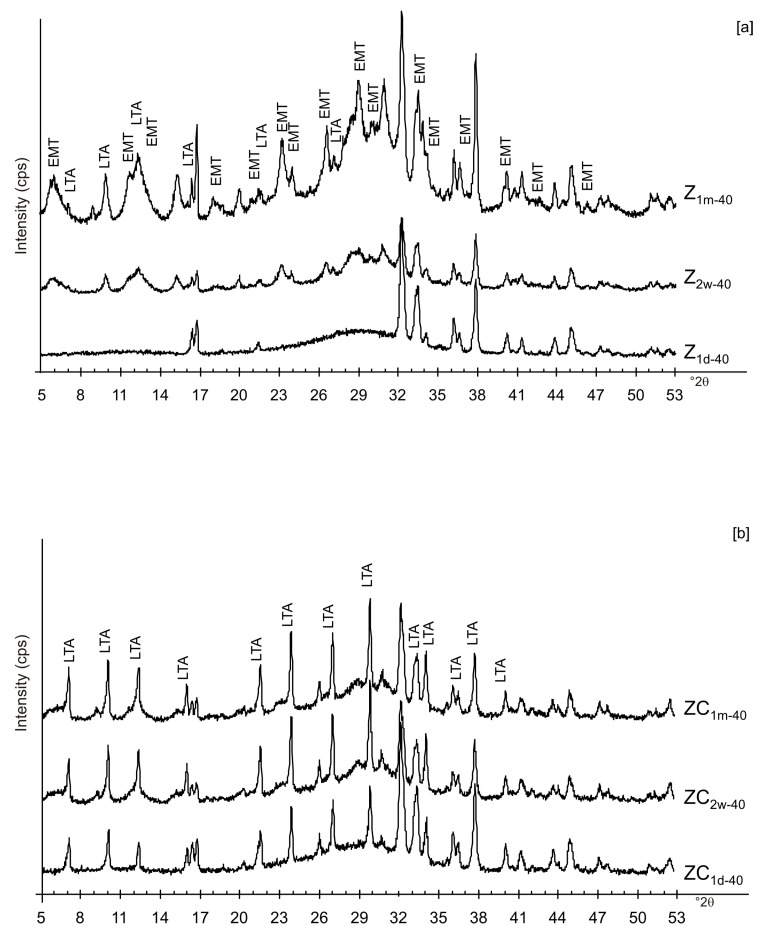
X-ray diffraction profiles of Z (**a**) and ZC (**b**) dried at 40 °C.

**Figure 10:**
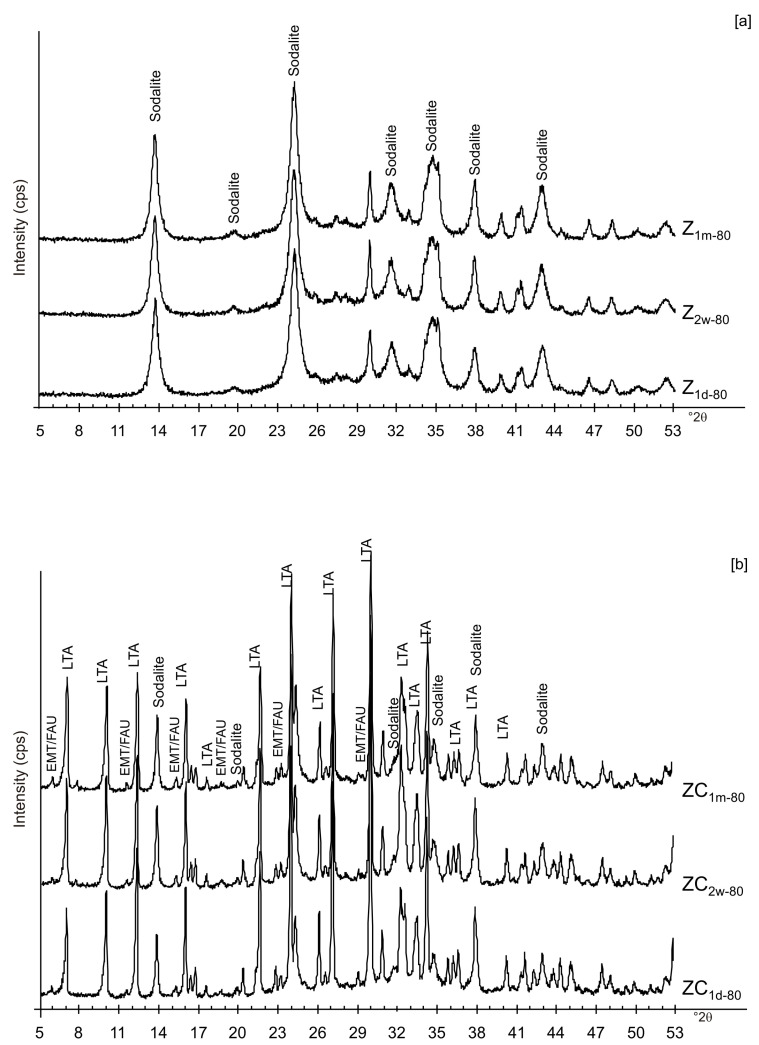
X-ray diffraction profiles of Z (**a**) and ZC (**b**) dried at 80 °C.

Figure 6a,b displays the XRD results for the samples dried at 80 °C and analysed after 2 weeks and 1 month of aging in the solid state. The data indicate that sodalite is the primary newly formed mineral in both CF and LN samples. XRD patterns of the samples washed with ethanol (LNE) show the absence of zeolite in all samples treated at 40 °C (Figure 7a) and the presence of sodalite after drying at 80 °C (Figure 7b and Figure 8). Finally, Figure 9 shows XRD profiles of ZC and Z samples at low drying temperature. The results demonstrate the synthesis of EMT in Z samples after 2 weeks and 1 month of aging. EMT is not present immediately after drying Z samples at 40 °C (Figure 9a). LTA is the primary newly formed zeolite in centrifuged samples (ZC) (Figure 9b). At higher temperature (80 °C), sodalite and LTA with sharp XRD peaks characterize Z and ZC products, respectively (Figure 10a,b).

The right-most column in Table 1 summarizes the mineralogical composition of each synthetic product.

## 4. Discussion

These preliminary results reveal the complex behavior of aluminosilicate precursor of LTA zeolite when subjected to combined freezing and drying processes. The data show that LTA zeolite does not form when the aluminosilicate precursor is rapidly frozen by liquid nitrogen and dried at 40 or 80 °C nor when it is dried at the same temperatures after conventional freezer treatment. These results suggest that the type of freezing treatment affects the availability of water in the precursor during the drying process (also based on the drying temperature), thereby influencing the type of zeolite formed and its stability after aging in the solid state. In detail, XRD profiles for both CF and LN immediately after drying at 40 °C are characterized by the presence of peaks indexed to aluminium silicate and sodium silicate. This is due to rapid precipitation in the supersaturated solution. Short time and lower heating temperature (40 °C) do not facilitate the synthesis of LTA zeolite (Figure 2a). This is also confirmed by the large presence of amorphous material. The data for the process at 80 °C show sodalite formation in both CF and LN samples (CF_1d-80_ and LN_1d-80_) (Figure 2b), and LTA zeolite does not form. SEM images are displayed in Figure 3a,b. After 2 weeks of aging in the solid state at room temperature, LTA zeolite did not form in CF samples initially dried at 40 °C (Figure 4a) whereas LN_2w-40_ is characterized by the presence of newly formed EMT zeolite detectable after 1 month in LN_1m-40_ (Figure 4b). This is well detectable in Figure 5a,b showing the typical EMT morphology. Both CF and LN samples that initially dried at higher temperature (80 °C) are characterized by the presence of sodalite (Figure 6a,b) after 2 weeks and 1 month of aging in the solid state.

We do not assume that the nature and the aggregation state of the aluminosilicate phases precipitated during the freezing process condition the behavior of the different samples. Our speculative hypothesis to explain the different behavior between CF and LN samples dried at a low temperature (40 °C), which is based on the assumption that rapid freezing by liquid nitrogen (−196 °C for few minutes) does not favor the nucleation of crystalline ice in the aluminosilicate precursor matrix, but, instead, leads to the formation of amorphous ice [29,40]. The presence of amorphous ice leads to a higher H_2_O activity in the matrix, which controls the transformation of aluminosilicate and amorphous material into EMT zeolite in 2 weeks (LN_2w-40_). Single crystals of sodalite also formed (Figure 5c). We propose that the process can be explained by considering partial hydrolysis at room temperature in the presence of enhanced H_2_O availability [56]. Conversely, the conventional freezer treatment is a slow process (2 h) occurring at −20 °C. This allows the formation of crystalline clusters [57] into the large amorphous material, thus making water molecules unavailable for zeolite crystallization at 40 °C and after aging in the solid state (CF_2w-40_ and CF_1m-40_). The presence of sodalite in both CF and LN samples dried at 80 °C (Figure 2b and Figure 6b) indicates that different processes take place, making water molecules available to form this more stable mineral. The presence of this newly formed phase after 2 weeks and 1 month of solid-state aging are in accordance with literature data [58,59]. Our hypothesis on the role of the H_2_O form (crystalline cluster or amorphous ice) in type and stability of zeolite formed is indirectly confirmed by additional experiments performed by washing the LN precursor with ethanol (before drying) or by repeating the synthesis without freezing. In the first case, the results indicate that, at 40 °C, no zeolite formed immediately after drying as well as after 2 weeks and 1 month of solid-state aging (LNE_1d-40_, LNE_2w-40_, and LNE_1m-40_) (Figure 7a). This is due to the partial replacement of water molecules by ethanol [60], confirming the importance of water availability in zeolite formation. After drying at 80 °C, sodalite formed (Figure 7b) despite the morphology of this newly formed mineral not being well organized, and the crystals are a few hundred nm in size (Figure 8).

Likely, the higher drying temperature makes the water molecules available, which are still confined in colloidal precursor after ethanol treatment. The data for untreated samples (no freezing or centrifugation) confirm the absence of zeolite immediately after drying at 40 °C. The progressive transformation of aluminosilicate and amorphous material into EMT and LTA takes place after 2 weeks and 1 month (Figure 9a). The results after centrifugation indicate the crystallization of LTA zeolite takes place at 40 °C, and LTA is stable over time (2 weeks and 1 month) (Figure 9b).

At 80 °C, sodalite formed in Z and is unchanged after solid-state aging (Figure 10a). These data are in accord with the results obtained at the same temperature for the LN sample. The results after centrifugation (ZC) reveal the crystallization of LTA zeolite at 80 °C as well as its presence after 2 weeks and 1 month (Figure 10b). Our hypothesis is that centrifugation has an influence on water available for the zeolite synthesis, conditioning LTA zeolite synthesis. Finally, no conclusion can be drawn on the effect of sonication.

## 5. Conclusions

In this study, the effects of freezing on zeolite synthesis from an LTA precursor was analysed with the aim to evaluate the role of H_2_O and its form as amorphous or crystalline ice in determining zeolite type and long-term stability.

Based on the results, our speculative hypothesis is that the formation of amorphous ice rather than crystalline ice in the aluminosilicate precursor controls the synthesis of different zeolites both immediately and after solid-state. The presence of amorphous ice in the precursor by a rapid liquid nitrogen freezing process (−196 °C) raises the possibility of the hydrolysis mechanism in the samples dried at 40 °C and zeolite evolution to a more stable form at room temperature. The formation of ice crystalline clusters by slow conventional freezer treatment at −20 °C, instead, is responsible for low water molecules availability in the samples, making no zeolite crystallization over time. In all the samples, the drying process of the precursor at 80 °C makes available water molecules due to a melting process of ice, forming sodalite with a stable behavior up to 1 month.

The results also display that the action of short time sonication after the freezing treatment is negligible.

In our opinion, these types of experiments make an important contribution to understanding crystallization mechanisms of minerals forming in extreme environmental conditions. However, further investigation is needed.

## Figures and Tables

**Table 1:** Scheme of Experiemnts and Relative Sample Codes.

Sample Code	Freezing Process		Additional Treatments		Drying Temperature		Aging Time at Solid State			Main Mineralogical Composition
	Liquid Nitrogen	Conventional Freezer	Ethanol Washing	Centrifuge	40 °C	80 °C	1 Day	2 Weeks	1 Month	
LN_1d-40_	x				x		x			Amorphous, Al and Na silicate
LN_1d-80_	x					x	x			Amorphous, Sodalite
LN_2w-40_	x				x			x		Amorphous, EMT, Sodalite
LN_2w-80_	x					x		x		Amorphous, Sodalite
LN_1m-40_	x				x				x	Amorphous, EMT, Sodalite
LN_1m-80_	x					x			x	Amorphous, Sodalite
CF_1d-40_		x			x		x			Amorphous, Al and Na silicate
CF_1d-80_		x				x	x			Amorphous, Sodalite
CF_2w-40_		x			x			x		Amorphous, Al and Na silicate
CF_2w-80_		x				x		x		Amorphous, Sodalite
CF_1m-40_		x			x				x	Amorphous, Al and Na silicate
CF_1m-80_		x				x			x	Amorphous, Sodalite
LNE_1d-40_	x		x		x		x			Amorphous, Al and Na silicate
LNE_1d-80_	x		x			x	x			Amorphous, Sodalite
LNE_2w-40_	x		x		x			x		Amorphous, Al and Na silicate
LNE_2w-80_	x		x			x		x		Amorphous, Sodalite
LNE_1m-40_	x		x		x				x	Amorphous, Al and Na silicate
LNE_1m-80_	x		x			x			x	Amorphous, Sodalite
ZC_1d-40_				x	x		x			Amorphous, LTA
ZC_1d-80_				x		x	x			LTA, EMT, sodalite
ZC_2w-40_				x	x			x		Amorphous, LTA
ZC_2w-80_				x		x		x		LTA, EMT, sodalite
ZC_1m-40_				x	x				x	Amorphous, LTA
ZC_1m-80_				x		x			x	LTA, EMT, sodalite
Z_1d-40_					x		x			Amorphous, Al and Na silicate
Z_1d-80_						x	x			Sodalite
Z_2w-40_					x			x		Amorphous, EMT, LTA
Z_2w-80_						x		x		Sodalite
Z_1m-40_					x				x	Amorphous, EMT, LTA
Z_1m-80_						x			x	Sodalite

## Supplementary Materials

The following are available online at https://www.mdpi.com/1996-1944/13/21/4780/s1. Figure S1: X-ray diffraction profiles of LN_2w-40_ and calculated EMT zeolite.

## References

[1] Baerlocher C., McCusker L.B. Database of Zeolite Structures. http://www.iza-structure.org/database/2008.

[2] Belviso C., Cavalcante F., Lettino A., Fiore S. (2013). A and X Type Zeolite Synthesized from Kaolinite at Low Temperature. Appl. Clay Sci..

[3] Rios C., Williams C., Fullen M. (2009). Nucleation and Growth History of Zeolite LTA Synthesized from Kaolinite by Two Different Methods. Appl. Clay Sci..

[4] Singh P., Aswal V.K., Chaudhri S.G., Schwieger W. (2018). Structural Evolution During Nucleation of Si-Rich LTA Nanocrystals from Colloidal Solution. Microporous Mesoporous Mater..

[5] Oleksiak M.D., Soltis J.A., Conato M.T., Penn R.L., Rimer J.D. (2016). Nucleation of FAU and LTA Zeolites from Heterogeneous Aluminosilicate Precursors. Chem. Mater..

[6] Belviso C., Agostinelli E., Belviso S., Cavalcante F., Pascucci S., Peddis D., Varvaro G., Fiore S. (2015). Synthesis of Magnetic Zeolite at Low Temperature Using a Waste Material Mixture: Fly Ash and Red Mud. Microporous Mesoporous Mater..

[7] Belviso C., Giannossa L.C., Huertas F.J., Lettino A., Mangone A., Fiore S. (2015). Synthesis of Zeolites at Low Temperatures in Fly Ash-Kaolinite Mixtures. Microporous Mesoporous Mater..

[8] Li Y., Peng T., Man W., Ju L., Zheng F., Zhang M., Guo M. (2016). Hydrothermal Synthesis of Mixtures of Na-A Zeolite and Sodalite from Ti-Bearing Electric Arc Furnace Slag. RSC Adv..

[9] Qian T., Li J. (2015). Synthesis of Na-A Zeolite from Coal Gangue With the in-Situ Crystallization Technique. Adv. Powder Technol..

[10] Fernandes-Machado N.R.C., Miotto D.M.M. (2005). Synthesis of Na–A and –X Zeolites from Oil Shale Ash. Fuel.

[11] Belviso S., Cavalcante F., Lettino A., Ragone P., Belviso C. (2016). Fly Ash as Raw Material for the Synthesis of Zeolite-Encapsulated Porphyrazine and Metallo Porphyrazine Tetrapyrrolic Macrocycles. Microporous Mesoporous Mater..

[12] Collins F., Rozhkovskaya A., Outram J.G., Millar G.J. (2020). A Critical Review of Waste Resources, Synthesis, and Applications for Zeolite LTA. Microporous Mesoporous Mater..

[13] Selim M.M., El-Mekkawi D.M., Aboelenin R.M.M., Sayed Ahmed S.A., Mohamed G.M. (2017). Preparation and Characterization of Na-A Zeolite from Aluminium Scrub and Commercial Sodium Silicate for the Removal of Cd^2+^ from Water. J. Assoc. Arab Univ. Basic Appl. Sci..

[14] Mintova S., Olson N.H., Valtchev V., Bein T. (1999). Nanocrystal Growth from Colloids at Room Temperature. Science.

[15] Ji Y., Zhang B., Zhao B., Li H., Wang N. (2017). High-efficient synthesis of zeolite LTA via a wet-gel crystallization route. Chem. Res. Chin. Univ..

[16] Smaïhi M., Barida O., Valtchev V. (2003). Investigation of the Crystallization Stages of LTA-Type Zeolite by Complementary Characterization Techniques. Eur. J. Inorg. Chem..

[17] Valtchev V.P., Tosheva L., Bozhilov K.N. (2005). Synthesis of Zeolite Nanocrystals at Room Temperature. Langmuir.

[18] Agger J.R., Pervaiz N., Cheetham A.K., Anderson M.W. (1998). Crystallization in Zeolite A Studied by Atomic Force Microscopy. J. Am. Chem. Soc..

[19] Yoon K.B. (2006). Mono and Multilayer Assembly of Zeolite Microcrystals on Substrates. Bull. Korean Chem. Soc..

[20] Yoon K.B. (2007). Organization of Zeolite Microcrystals for Production of Functional Materials. Acc. Chem. Res..

[21] Leiggener C., Calzaferri G. (2004). Monolayers of Zeolite A Containing Luminescent Silver Sulfide Clusters. Chem. Phys. Chem..

[22] Walton R.I., Millange F., O’Hare D., Davies A.T., Sankar G., Catlow C.R.A. (2001). An In Situ Energy-Dispersive X-ray Diffraction Study of the Hydrothermal Crystallization of Zeolite A. 1. Influence of Reaction Conditions and Transformation into Sodalite. J. Phys. Chem. B.

[23] Subotic B., Sekovanic L. (1986). Transformation of Zeolite A into Hydroxysodalite. J. Cryst. Growth.

[24] Greer H., Wheatley P.S., Ashbrook S.E., Morris R.E., Zhou W. (2009). Early Stage Reversed Crystal Growth of Zeolite A and Its Phase Transformation to Sodalite. J. Am. Chem. Soc..

[25] Belviso C. (2018). Ultrasonic vs Hydrothermal Method: Different Approaches to Convert Fly Ash into Zeolite. How They Affect the Stability of Synthetic Products Over Time?. Ultrason. Sonochemistry.

[26] Belviso C., Lettino A., Cavalcante F. (2018). Influence of Synthesis Method on LTA Time-Dependent Stability. Molecules.

[27] Cundy C.S., Cox P.A. (2003). The Hydrothermal Synthesis of Zeolites: History and Development from the Earliest Days to the Present Time. Chem. Rev..

[28] Bushuev Y., Sastre G., De Julián-OrtizJ V. (2009). The Structural Directing Role of Water and Hydroxyl Groups in the Synthesis of Beta Zeolite Polymorphs. J. Phys. Chem. C.

[29] Demontis P., Gulín-González J., Jobic H., Masia M., Sale R., Suffritti G.B. (2008). Dynamical Properties of Confined Water Nanoclusters: Simulation Study of Hydrated Zeolite Na-A: Structural and Vibrational Properties. ACS Nano.

[30] Angell C.A. (2008). Insights into Phases of Liquid Water from Study of Its Unusual Glass-Forming Properties. Science.

[31] Marcolli C. (2014). Deposition Nucleation Viewed as Homogeneous or Immersion Freezing in Pores and Cavities. Atmos. Chem. Phys. Discuss..

[32] Jelassi J., Castricum H., Bellissent-Funel M.-C., Dore J., Webber J.B.W., Sridi-Dorbez R. (2010). Studies of Water and Ice in Hydrophilic and Hydrophobic Mesoporous Silicas: Pore Characterisation and Phase Transformations. Phys. Chem. Chem. Phys..

[33] Seyed-Yazdi J., Farman H., Dore J.C., Webber J.B.W., Findenegg G.H. (2008). Structural Characterization of Water/Ice Formation in Sba-15 Silicas: III. the Triplet Profile for 86 Å Pore Diameter. J. Phys. Condens. Matter.

[34] Liu E., Dore J.C., Webber J.B.W., Khushalani D., Jähnert S., Findenegg G.H., Hansen T. (2006). Neutron Diffraction and NMR Relaxation Studies of Structural Variation and Phase Transformations for Water/Ice in SBA-15 Silica: I. the Over-Filled Case. J. Phys. Condens. Matter.

[35] Jażdżewska M., Sliwinska-Bartkowiak M., Domin K., Chudoba D.M., Beskrovnyi A.I., Neov D.S., Gubbins K.E. (2019). Structure of Ice Confined in Carbon and Silica Nanopores. Bull. Mater. Sci..

[36] Bordonskii G.S., Orlov A.O. (2019). Phase Transitions of Water in Zeolite Pores. Tech. Phys. Lett..

[37] Limmer D.T., Chandler D. (2012). Phase Diagram of Supercooled Water Confined to Hydrophilic Nanopores. J. Chem. Phys..

[38] Menshikov L.I., Menshikov P.L., Fedichev P.O. (2017). Phenomenological Model of Hydrophobic and Hydrophilic Interactions. J. Exp. Theor. Phys..

[39] Janssen A.H., Talsma H., Van Steenbergen M.J., De Jong K.P. (2004). Homogeneous Nucleation of Water in Mesoporous Zeolite Cavities. Langmuir.

[40] Kyakuno H., Matsuda K., Nakai Y., Fukuoka T., Maniwa Y., Nishihara H., Kyotani T. (2013). Amorphous Water in Three-Dimensional Confinement of Zeolite-Templated Carbon. Chem. Phys. Lett..

[41] Jiang Z., Zhao G., Hossain S.M.C., Gao D. (2017). Coupled Experimental-Modeling Analyses of Heat Transfer in Ex-Vivo VS55-Perfused Porcine Hepatic Tissue Being Plunged in Liquid Nitrogen for Vitreous Cryopreservation. Int. J. Heat Mass Transf..

[42] Fahy G., Macfarlane D., Angell C., Meryman H. (1984). Vitrification as an Approach to Cryopreservation. Cryobiology.

[43] Faizullin M.Z., Koverda V.P. (2012). Vitrification and Crystallization of Low-Temperature Amorphous Condensates of Water and Methane—Water Mixture. Russ. J. Phys. Chem. A.

[44] Poudyal R.L., Kobayashi R., Suzuki T., Watanabe M. (2019). Effect of Different Freezing and Storage Condition on the Physical Properties of Protein Coagulum (Firm Tofu). Int. J. Refrig..

[45] Olmo A., Baena R., Risco R. (2008). Use of a Droplet Nucleation Analyzer in the Study of Water Freezing Kinetics Under the Influence of Ultrasound Waves. Int. J. Refrig..

[46] Kiani H., Zhang Z., Sun D.-W. (2013). Effect of Ultrasound Irradiation on Ice Crystal Size Distribution in Frozen Agar Gel Samples. Innov. Food Sci. Emerg. Technol..

[47] Dalvi-Isfahan M., Hamdami N., Xanthakis E., Le-Bail A. (2017). Review on the Control of Ice Nucleation by Ultrasound Waves, Electric and Magnetic Fields. J. Food Eng..

[48] Cheng X., Zhang M., Xu B., Adhikari B., Sun J. (2015). The Principles of Ultrasound and Its Application in Freezing Related Processes of Food Materials: A Review. Ultrason. Sonochemistry.

[49] Inaba H., Takeya K., Nozu S. (1994). Fundamental Study on Continuous Ice Making Using Flowing Supercooled Water. JSME Int. J. Ser. B.

[50] Simonetti F., Fox M. (2019). Experimental Methods for Ultrasonic Testing of Complex-Shaped Parts Encased in Ice. NDT E Int..

[51] Mohd Khairi M.T., Ibrahim S., Md Yunus M.A., Faramarzi M., Abid A. (2019). Experimental Methods for Ultrasonic Testing of Complex-Shaped Parts Encased in Ice. Measurement.

[52] Saclier M., Peczalski R., Andrieu J. (2010). Effect of Ultrasonically Induced Nucleation on Ice Crystals’ Size and Shape During Freezing in Vials. Chem. Eng. Sci..

[53] Inada T., Zhang X., Yabe A., Kozawa Y. (2001). Active Control of Phase Change from Supercooled Water to Ice by Ultrasonic Vibration 1. Control of Freezing Temperature. Int. J. Heat Mass Transf..

[54] Gao P., Zhou X., Cheng B., Zhang D., Zhou G. (2017). Study on Heat and Mass Transfer of Droplet Cooling in Ultrasound Wave. Int. J. Heat Mass Transf..

[55] Thompson R.W., Huber M.J. (1982). Analysis of the Growth of Molecular Sieve Zeolite NaA in a Batch Precipitation System. J. Cryst. Growth.

[56] Heard C.J., Grajciar L., Rice C.M., Pugh S.M., Nachtigall P., Ashbrook S.E., Morris R.E. (2019). Fast Room Temperature Lability of Aluminosilicate Zeolites. Nat. Commun..

[57] Wang R., Gouseti O., Lopez-Quiroga E., Fryer P.J., Bakalis S. (2019). Water Crystallization in Highly Concentrated Carbohydrate-Based Systems. Cryst. Growth Des..

[58] Breuer R.G., Barsotti L.R., Kelly A.C. (1963). Behaviour of Silica in Sodium Aluminate Solutions. International Symposium on the Extract Metallurgy of Aluminium.

[59] Liu Q., Navrotsky A. (2007). Synthesis of Nitrate Sodalite: An in Situ Scanning Calorimetric Study. Geochim. Cosmochim. Acta.

[60] Li X., Wang X., Passaro M.D., Spinelli N., Apicella B. (2015). Insights on Clusters Formation Mechanism by Time of Flight Mass Spectrometry. 1. The Case of Ethanol–Water Clusters. J. Am. Soc. Mass Spectrom..

