# Supplementary material for: Effect of H2O Activity on Zeolite Formation
Source: Materials (Basel). 2020 Oct 26;13(21):4780. doi: 10.3390/ma13214780 (PMC7662609; doi:10.3390/ma13214780)
Supplement: Supplementary file 1 [file materials-13-04780-s001.pdf]

Supplementary Materials

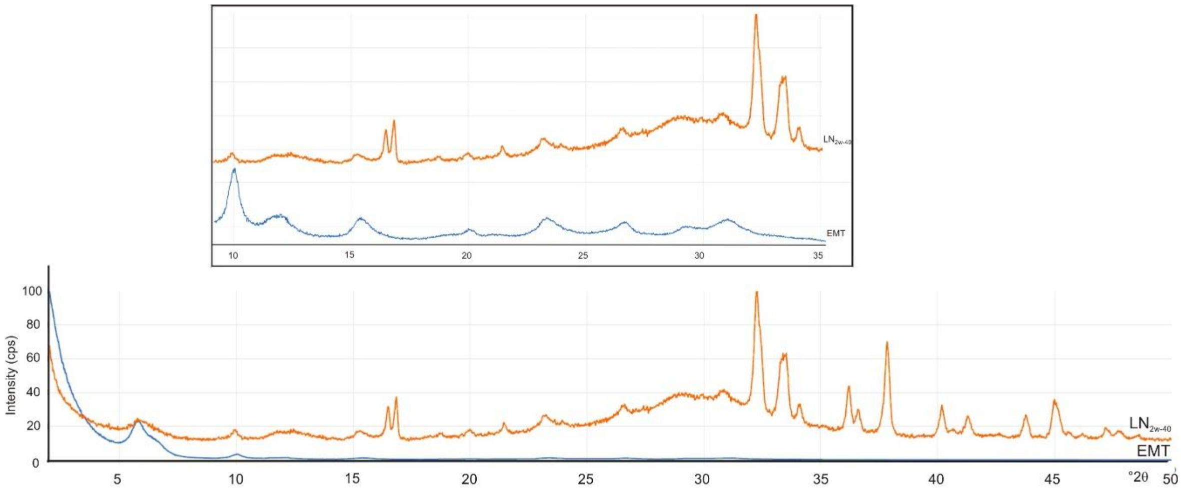

Figure S1. X-ray diffraction profiles of LN_{2w-40} and calculated EMT zeolite.
